# The Impact of Adaptive Learning in Entrepreneurial Behavior for College Students

**DOI:** 10.3389/fpsyg.2021.797459

**Published:** 2022-02-22

**Authors:** Dan Yang

**Affiliations:** School of Graduate Studies, Lingnan University, Tuen Mun, Hong Kong SAR, China

**Keywords:** college students’ entrepreneurship, epidemic era, structural equation modeling, entrepreneurial behavior choice, entrepreneurial ability

## Abstract

Entrepreneurship of college students has always been a hot topic in families, schools and society. Massive studies aim to explore entrepreneurial behavior. However, under the condition of the 10% success rate of student entrepreneurship, the adverse impact of COVID-19 and the changed circumstance of domestic entrepreneurship, this exploration aims to study the factors that influence college students’ entrepreneurial behavior choices under the epidemic. First, through the retrieval of relevant literature and theoretical study, the variable factors that affect behavior choices are sorted and summarized. It is assumed that the factors that affect behavior choices are entrepreneurial motivation, entrepreneurial ability, willingness to behave, and entrepreneurial environment. Second, a questionnaire is designed to investigate the choice of entrepreneurial behavior for students who are starting a business or going to start a business. The standard effect values of the survey results are calculated by using structural equation modeling (SEM). The results reveal that the effect values of the nine hypothetical results are all in line with the prediction, which prove a positive impact of the four variable factors on the choice of entrepreneurial behavior. The experimental parameters set are as follows. The standardized effect value of Hypothesis 1 (entrepreneurial motivation has a positive impact on entrepreneurial behavior choice) is 0.216; that of Hypothesis 2 (entrepreneurial ability has a positive impact on the choice of entrepreneurial behavior) is 0.221; that of Hypothesis 3 (willingness to behave has a positive impact on entrepreneurial behavior choice) is 0.284; that of Hypothesis 4 (entrepreneurial environment has a positive impact on the choice of entrepreneurial behavior) is 0.329; that of Hypothesis 5 (entrepreneurial motivation has a positive impact on entrepreneurial intention) is 0.247; that of Hypothesis 6 (entrepreneurial ability has a positive impact on willingness to behave) is 0.339; that of Hypothesis 7 (entrepreneurial ability has a positive impact on entrepreneurial motivation) is 0.357; that of Hypothesis 8 (entrepreneurial environment has a positive impact on willingness to behave) is 0.336; that of Hypothesis 9 (entrepreneurial environment has a positive impact on entrepreneurial motivation) is 0.485. Besides, the entrepreneurial environment has the greatest impact on behavior choice. Therefore, it is believed that the government, society, schools need to strengthen the correct guidance of entrepreneurial students and create a good entrepreneurial environment to cope with economic changes under the epidemic.

## Introduction

Since the 18th National Congress of the Communist Party of China, the state has encouraged and supported college students to start their own businesses. College students from all over the country have joined the entrepreneurial trend, but the result is not optimistic (Wenfeng, 2020). As the COVID-19 swept across the cities of China at the end of 2019, the central government has promulgated strong prevention and control policies one after another, and the epidemic was finally overcome with the efforts of whole nation. However, many small and medium-sized enterprises have faced bankruptcy due to the 4-month shutdown in China, the current situation of the epidemic abroad, and the impact of uncertain factors of world trade on the economic system (Wang and Hu, 2021), so that the immature college students are faced with many risks and fall into a dilemma.

Nowadays, there are not enough jobs to satisfy everyone, so the original intention of college students to start a business is to alleviate the employment pressure. Yong et al. (2020) analyzed the current situation, hotspots and frontiers of the employment pressure research of college students. The result suggested that college students’ employment pressure will be affected by social support, mental health education and coping methods (Yong et al., 2020). During the epidemic period, how to promote college students’ entrepreneurship is an issue that the state needs to pay more attention to. Wang et al. (2020) believed that exploratory and developmental learning will play a key role in enterprise growth performance. Therefore, it is necessary to improve the innovation of college students’ entrepreneurship. Colleges need to guide students’ entrepreneurship according to the situation of students. Wu et al. (2017) mentioned in the research that educators and policy makers should integrate the views of function, personality and behavior into the acceptance of entrepreneurship, so as to affect a country’s economic development. Besides, in the process of entrepreneurship, students’ personal psychological quality will also play a key role in entrepreneurship. Zheng et al. (2019) found that narcissism will have a negative impact on entrepreneurship. Finally, Song and Wu (2019) found that good social style and trust between partners will also have an impact on entrepreneurship. However, due to the outbreak of the epidemic, students are facing great pressure to start a business. Although entrepreneurial enthusiasm is high, the success rate is always no more than 10%.

In recent years, the research mainly focuses on the entrepreneurial behavior of college students. There are more studies on entrepreneurial models and entrepreneurial projects, but few on the factors affecting entrepreneurship. After the outbreak of the epidemic, it is necessary to analyze a series of factors affecting students’ entrepreneurial behavior. Therefore, this exploration aims to study the influencing factors of entrepreneurial environment on college students’ entrepreneurship after the epidemic. First, relevant literature and analyses about the influencing factors of college students’ entrepreneurship are collected. Then, a questionnaire on the entrepreneurial environment and entrepreneurial ability of college students who have started or will start a business is made. Finally, the data analysis software SPSS26.0 (statistical product and service solutions) and the analysis algorithm structural equation modeling (SEM) are used to test the reliability and validity of the survey results to verify the correlation between variables. The innovation is to analyze the behavioral influencing factors of entrepreneurs’ entrepreneurial behavior.

## Literature Review

Many studies have confirmed the impact of the epidemic on entrepreneurs’ willingness. Ting et al. (2021) conducted a multi factor analysis on the opportunities for the sustainable development of professional sports enterprises and events from the perspective of public awareness, attitude and behavior, as well as the physical and mental health of spectators of professional events in Taiwan. It aimed to maintain the normal operation of professional sports companies and events under the COVID-19, and create a sustainable development environment for them (Ting et al., 2021). Cao and Wang (2020) found that since the COVID-19 pandemic, China’s higher vocational college students have encountered great problems in finding jobs. They believed that efforts should be made to improve the employment environment to improve students’ attitude, self-quality and personal entrepreneurial interest. In addition, government policies and family support were considered to assess the impact of external support on students’ decisions. The results show that students’ attitude, self-quality, and personal interest will affect their entrepreneurial intention. The impact of family support is greater than that of government policy. Government policies have no significant impact on students’ entrepreneurial intention (Cao and Wang, 2020). The above two studies theoretically show that the epidemic has a significant impact on entrepreneurial intention. Given the significant impact of new enterprises on the global economy, Murnieks et al. (2020) understood the motivation of entrepreneurs, which had practical and theoretical importance. It could make people understand how motivation drives the start-up, growth and exit of enterprises. The existing literature was sorted out and reviewed based on the entrepreneurial motivation of each stage of the new entrepreneurial process. Therefore, the research status of entrepreneurial motivation and the road map of its rule network were formulated, and suggestions were provided to guide future research, so as to expand people’s understanding of motivation in the field of entrepreneurship and traditional organizational environment (Murnieks et al., 2020). Allmendinger and Berger (2020) tried to understand when the founders of new enterprises are willing to participate in this asymmetric partnership by considering the characteristics of entrepreneurial decision-makers and the perceived attributes of larger competitors. The research shows that entrepreneurs’ self-efficacy reduces the positive impact of cooperation intention and concise contract design. The results have an impact on the self-concept and design of innovation and partner management of large companies, as well as entrepreneurs who regard asymmetric partnerships as growth opportunities (Allmendinger and Berger, 2020). Through experiments, these experts proved that individual entrepreneurial opportunities are affected by entrepreneurial environment and entrepreneurial intention, and the impact of entrepreneurial environment is the most significant. Boahemaah et al. (2020) studied the impact of entrepreneurship intention education on college students through personal factors (attitude toward behavior, entrepreneurial motivation, entrepreneurial resources and perceived behavior control). The results reveal that individual factors and entrepreneurship education have a direct positive impact on entrepreneurship intention (Boahemaah et al., 2020). Caliendo et al. (2020) analyzed the impact of personal level (human capital and personality) and the impact of business-related characteristics on these two indicators and their relative importance. It shows that the results are heterogeneous between groups. In particular, the previously founders have no difference in survival opportunities, but they are more likely to lack a high psychological commitment to entrepreneurship (Caliendo et al., 2020). Through the experiment, the specific influencing factors of entrepreneurial motivation were predicted, and the experimental results were verified, which further shows that the entrepreneurial environment exerts a great impact on entrepreneurial motivation. Therefore, this exploration will take entrepreneurial environment as the main factor to explore the influencing factors of COVID-19’s entrepreneurial motivation.

## Materials and Methods

### College Students’ Entrepreneurship

Due to the transformation of the market economy and the increase of employment pressure, entrepreneurship, as a free way of career choice, has been favored by young people (Zhang, 2020). College students’ entrepreneurship is the social behavior of college students or graduates to create independent enterprises. However, it has both advantages and disadvantages. To provide support for college students’ entrepreneurship, many scholars believe that students must improve their comprehensive ability, schools should pay attention to the cultivation of entrepreneurial ability, and society must focus more on students’ entrepreneurship.

**FIGURE 1:**
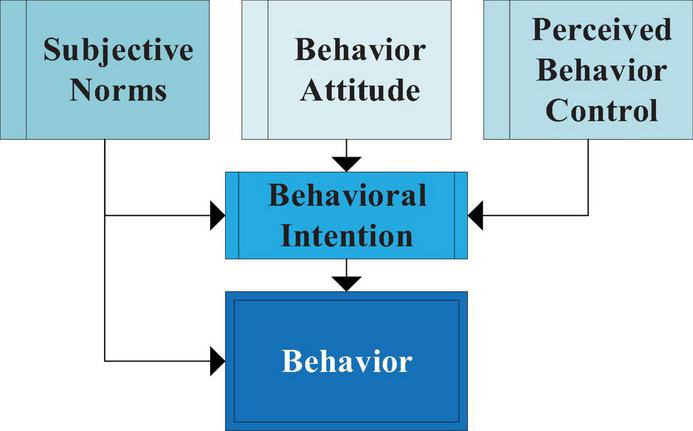
The model of planned behavior theory.

Entrepreneurial behavior comes from the west, not only the operation of large-scale organizations, but also the groups preparing to set up new enterprises. The concept of planned behavior theory is a result of individual thinking. It can help people understand how individuals change their behavior, so as to explain and predict individual behavior more appropriately. Planned behavior is a perfect concept of social psychology and has been applied in the world (Schmidt and Stenger, 2021). Figure 1 displays its theoretical structure.

**FIGURE 2:**
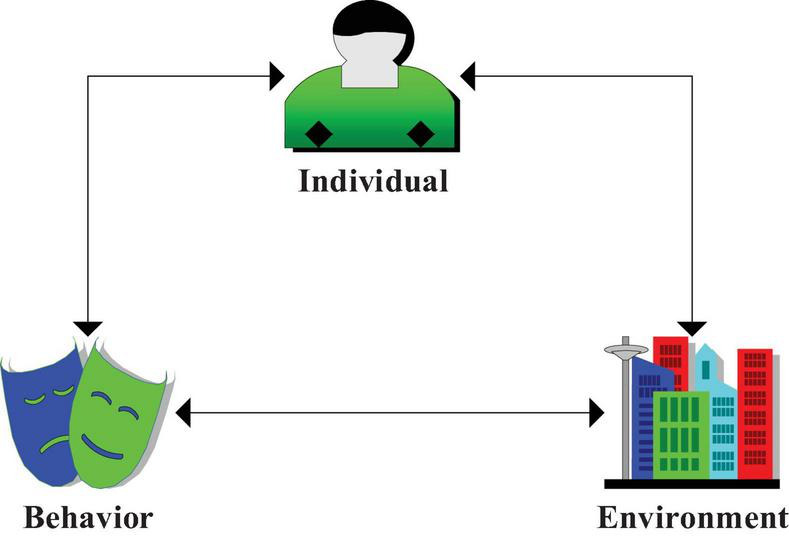
The model of ternary interaction theory.

Ternary interaction theory is a theory that describes the interaction among the individual, behavior and environment. The three elements interact and act as cause and effect, which determine the occurrence and realization of various behaviors together. The individual elements in the ternary interaction theory can trigger individuals to perceive and understand other internal characteristics, including personal IQ, personality, confidence, attitude, and view of things (Feng, 2020). It also includes physiological characteristics, race, temperament and heredity. The environment is unstable and generally affects people through behavior. The three coexist and promote together. Figure 2 shows the structure.

**FIGURE 3:**
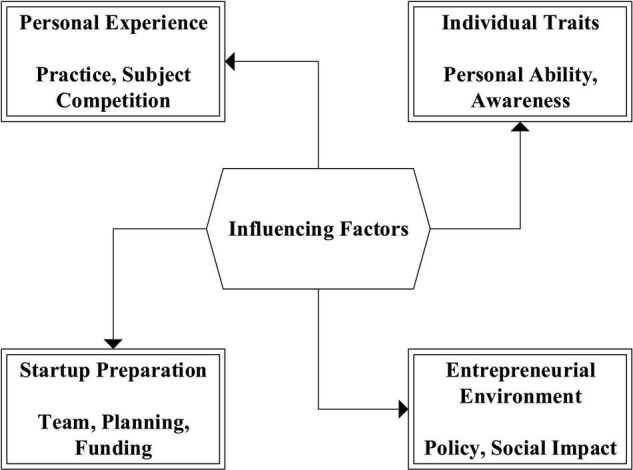
The influencing factors of entrepreneurial choice.

In recent years, the research on the choice of college students’ entrepreneurial behavior has increased. The research has found that most college students choose the tertiary industry, so few will get official help and venture capital. There are great differences in the industry, enterprise scale and capital source of college students’ entrepreneurship choice. Some scholars believe that the choice of entrepreneurship has certain rules, among which the most influential factors are funds, prospects, environment and competitiveness. However, the others believe that personal personality is the main influencing factor (Vodǎ and Florea, 2019), followed by family, professional restrictions and gender (Hutasuhut, 2018; Wang et al., 2018). Some studies show that entrepreneurs have summarized four influencing factors from the entrepreneurial process. Figure 3 displays the four influencing factors.

### Hypothesis of Influencing Factors of College Students’ Entrepreneurship Choice Behavior

**FIGURE 4:**
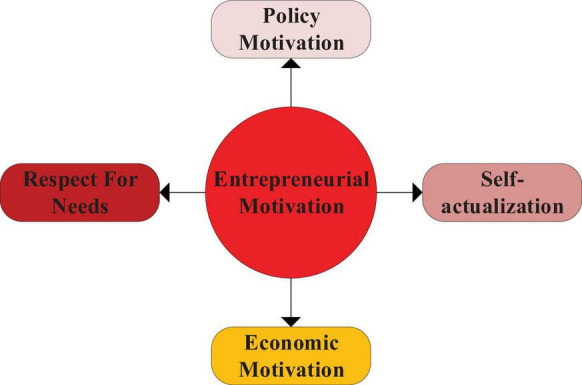
Variable indicators of entrepreneurial motivation.

**FIGURE 5:**
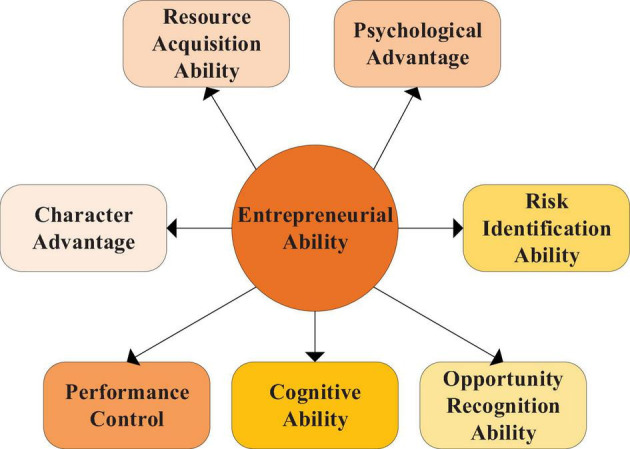
Variable indicators of entrepreneurial ability.

**FIGURE 6:**
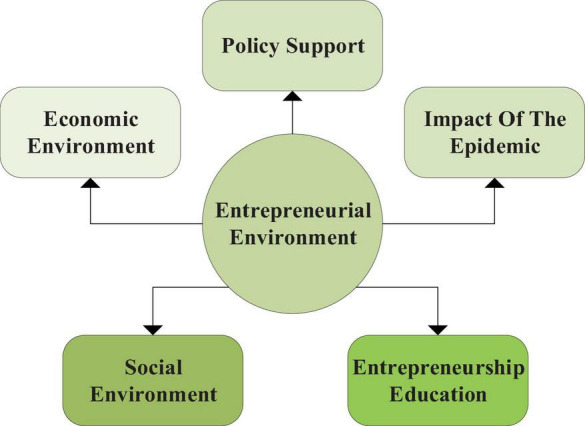
Variable indicators of entrepreneurial environment.

Based on the factors affecting entrepreneurial choice behavior studied by previous scholars, the impact of social changes on entrepreneurial choice behavior after the epidemic is analyzed, and the variable factors according to the concept of planned behavior and ternary interaction theory are assumed. Through literature review and previous research results, it is considered that the choice of entrepreneurial behavior of college students is restricted by many factors, and this epidemic has brought these influencing factors into play, which has a greater impact on entrepreneurs’ motivation and environment. Therefore, this exploration aims to analyze the influencing factors in the epidemic environment and summarize the commonality and build the selection influencing factor model. Among the subjective factors, there are three aspects: entrepreneurial motivation, entrepreneurial ability and willingness to behave. Each of the three aspects includes several influencing factors:

(1) Entrepreneurial motivation: It is the motivation and purpose of entrepreneurs’ entrepreneurial behavior and affects entrepreneurs’ behavioral enthusiasm (Mahto and McDowell, 2018). Both internal motivation and external motivation can stimulate, maintain and adjust the entrepreneurial level. They are major influencing factors of entrepreneurs’ behavior, including policy motivation, economic motivation, respect for needs and self-actualization. Figure 4 shows the structure.

(2) Entrepreneurial ability: it is reflected in entrepreneurs’ learning, cognition, innovation and practice, which is a necessary quality for an enterprise to succeed (Sun et al., 2020). Other comprehensive qualities can bring advantages in entrepreneurship. Combining entrepreneurial quality and entrepreneurial ability, this exploration aims to analyze the influencing factors of students’ entrepreneurial behavior choice according to the impact of the epidemic on entrepreneurs themselves and the attitude of entrepreneurs toward the epidemic, and analyze entrepreneurial ability from the aspects of resource acquisition ability, risk identification ability, character advantage, psychological advantage, opportunity recognition ability and performance control. Figure 5 shows the structure and content.

(3) Willingness to behave: willingness is a kind of emotional state of people essentially, which is a designated goal or measure that people face when they want to get something (Tentama, 2018). Behavior is the action made by people according to their psychological instructions, which can express people’s attitudes. In the process of entrepreneurship, college students can analyze the impact of the environment in terms of entrepreneurial goals, decisions, and actions. The research indicators of behavior intention here are the degree of willingness to behave.
(4) Objective factors mainly refer to the entrepreneurial environment. The impact of the external environment on entrepreneurship includes public opinion and policy support, including the economic environment (Boudreaux et al., 2019), social environment, entrepreneurship education (Ndofirepi, 2020), policy support and impact of the epidemic (Lei et al., 2020). Figure 6 shows its structure.

Result hypothesis: based on the above factors affecting the choice of entrepreneurial behavior and the cognition of relevant research results, the hypothesis of research results is made.

H1: entrepreneurial motivation has a positive impact on entrepreneurial behavior choice: good entrepreneurial motivation can stimulate entrepreneurs’ enthusiasm and potential, which is conducive to achieving entrepreneurs’ goal of the behavior choice.

H2: entrepreneurial ability has a positive impact on the choice of entrepreneurial behavior: The strength of entrepreneurial ability can directly affect the initiative and rationality of entrepreneurial behavior. College students with entrepreneurial ability can strengthen their self-confidence, and entrepreneurs with strong ability can make more excellent behavior choices in a complex environment in the case of an epidemic.

H3: willingness to behave has a positive impact on entrepreneurial behavior choice: the entrepreneurs’ willingness to behave is the subjective performance of their upcoming or ongoing entrepreneurship. The strength of choice intention marks the influence degree of the behavior choice. Combined with the impact of the epidemic, it is considered that entrepreneurs with strong behavioral willingness will help them make entrepreneurial behavior choices.

H4: entrepreneurial environment has a positive impact on the choice of entrepreneurial behavior: the entrepreneurial environment is the impact of the external environment of college students’ entrepreneurship, including economic, social and policy. The integration of college students’ individuals and entrepreneurial environment is to produce entrepreneurial behavior.

H5: entrepreneurial motivation has a positive impact on entrepreneurial intention: some scholars believe that entrepreneurial motivation has a significant impact on behavior intention and it is a factor of entrepreneurs’ understanding of social rules.

H6: entrepreneurial ability has a positive impact on willingness to behave: the study of entrepreneurial motivation cannot omit the entrepreneur’s own ability. Entrepreneurs will have different entrepreneurial motivations according to their own abilities. Therefore, the entrepreneurial ability will also have an impact on willingness to behave.

H7: entrepreneurial ability has a positive impact on entrepreneurial motivation: similar to H6, the strength of ability will change entrepreneurs’ judgment of entrepreneurial motivation.

H8: entrepreneurial environment has a positive impact on willingness to behave: the quality of entrepreneurial environment will affect entrepreneurs’ own understanding of entrepreneurship. A good environment will encourage college students’ willingness, while a bad environment will inhibit college students’ entrepreneurial intention.

H9: entrepreneurial environment has a positive impact on entrepreneurial motivation: social, family and school environments will stimulate entrepreneurs’ psychology. The more favorable the environment is, the stronger the entrepreneurial motivation will be.

**FIGURE 7:**
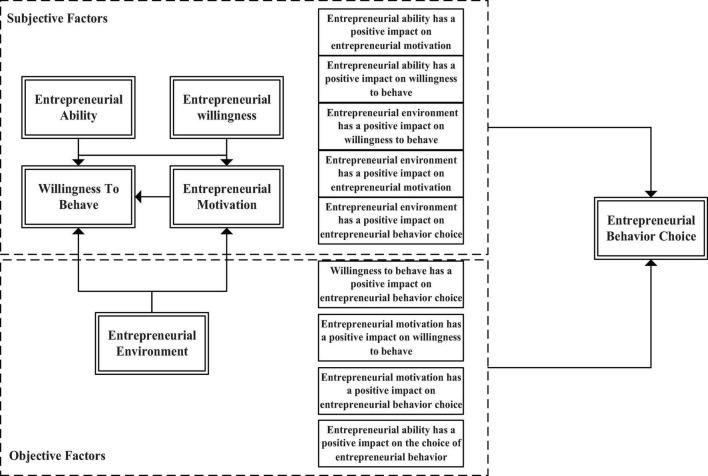
The influencing factor model of the entrepreneurial behavior choice.

According to the summarized factors affecting the choice of entrepreneurial behavior, the relationship among variables is connected in series, and the influence model of entrepreneurial behavior choice is constructed on the premise of the epidemic situation, as shown in Figure 7.

### Questionnaire on Entrepreneurial Behavior of College Students

The questionnaire can show the influencing factors of college students’ entrepreneurial behavior choice in the epidemic era truly and accurately. First, the theories about college students’ entrepreneurship on platforms such as college students’ entrepreneurship competition or entrepreneurship work group are collected. Then, the first draft of the questionnaire is designed. Besides, the questionnaire is modified according to the actual situation. In the questionnaire, except for the personal information of the respondents, Likert grade 5 scoring scale (Park and Wu, 2019) is used for other questions. The questionnaire includes 5 variables of motivation, ability, willingness, environment and choice, and 26 corresponding observation variables as well. Table 1 shows the initial scale.

**TABLE 1:** The initial scale of influencing factors.

	Variable	Code	Questions
Basics	Personal information (BI1-8)	BI1	Gender
		BI2	Age
		BI3	Educational background
		BI4	Professional type
		BI5	Number of entrepreneurial experiences
		BI6	The type of industry you are starting a business or about to start a business.
		BI7	The model you are starting or about to start.
		BI8	Scale of entrepreneurship
Subjective reasons (motivation, ability, willingness)	Entrepreneurial motivation (EM1-4)	EM1	Recognized by the society
		EM2	Accumulate wealth
		EM3	Respond to government requirements
		EM4	Realize personal wishes
	Entrepreneurial ability (ERC1-9)	ERC1	Stable financial and technical support
		ERC2	Able to take risks
		ERC3	Cheerful personality
		ERC4	Positive attitude toward work and life
		ERC5	Good business sense
		ERC6	Clear self-awareness
		ERC7	Understand the requirements for entrepreneurship
		ERC8	Strong management ability
		ERC9	Hope to create performance
	Willingness to behave (BI1-3)	BI1	Change through behavioral choice
		BI2	The choice is important for entrepreneurship.
		BI3	Changes must be made.
Scenario reason (environment)	Entrepreneurial environment (EE1-5)	EE1	The domestic situation is suitable for entrepreneurship.
		EE2	Entrepreneurship is not affected by the epidemic.
		EE3	With the support of relatives and friends
		EE4	Received entrepreneurship education in school.
		EE5	Entrepreneurship policy helps a lot.
Dependent variable (choice)	Entrepreneurial choice (EC1-5)	EC1	Have confidence in the choice.
		EC2	The entrepreneurial process is relatively smooth.
		EC3	The entrepreneurial intention is clear.
		EC4	Able to make choices easily
		EC5	The original intention of starting a business meets expectations.

Reliability analysis of the questionnaire: the higher the reliability of the questionnaire is, the more accurate the actual situation is, and the more convincing the results of the questionnaire will be. Among the reliability analysis methods, the most widely used method is to use Cronbach’s α coefficient to calculate its reliability, and its equation is:


(1) $$A\,l\,p\,h\,a=\left(\frac{k}{k-1}\right)\,\left(1-\frac{\sum_{i=1}^{k}S_{i}^{2}}{S_{x}^{2}}\right)$$

In (1), *k* represents the total number of questions, $S_{i}^{2}$represents the variance of the score of the i-th item, and $S_{x}^{2}$represents the variance of the total number of items. It can be seen that the α coefficient can be used to measure the unity of attitudes and opinions.

The algorithm of SEM (Carden et al., 2019; Dwivedi et al., 2021) has the function of verification. It is an extension of the general linear model, which can make up for the shortcomings of traditional statistics and analyze multiple variables and results. The SEM generally requires that the sample size is greater than 100, and the verification effect is the best if it is more than 200. To meet *N/P* > 10 and *N/t* > 5, *N* is the total sample capacity, *t* is the number of free parameters, and *P* is the total observed variable. Equation (2) displays the SEM equation:


(2) η=β⁢η+γ⁢ξ+ζ

η represents the internal dependent variable, ξ represents the external dependent variable, and ζ represents the residual term of the structural equation. Equations of the measurement model are:


(3) $$X=\wedge_{x}\,\xi +\delta$$

(4) $$Y=\wedge_{y}\,\eta +\epsilon$$

Among them, *X* and *Y* represent the two observed variables for calculating the internal and external dependent variables η and ξ, δ and ϵ are the errors of *X* and *Y* measurement, ∧_*x*_ is the load on the *X* and ξ variables, and ∧_*y*_ is the load on the η variable.

**FIGURE 8:**
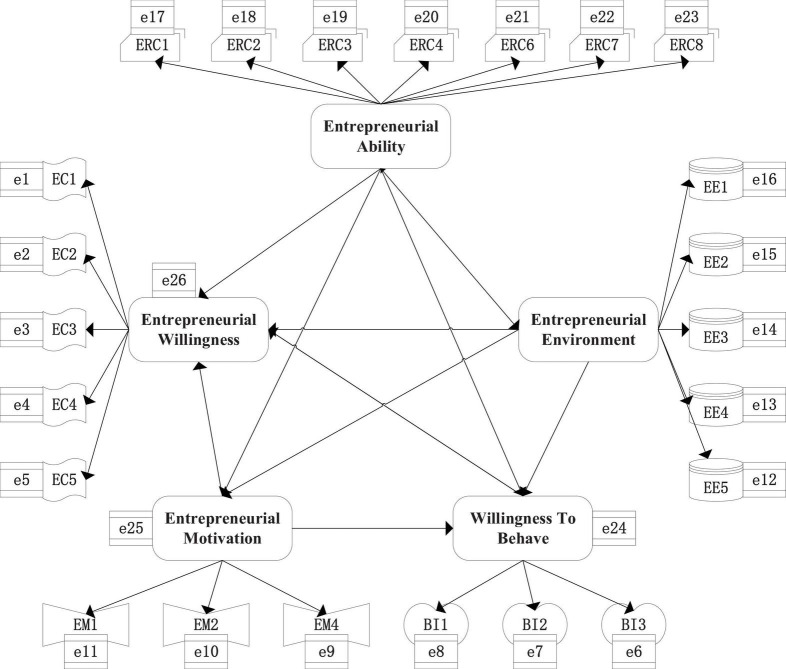
SEM | The final questionnaire: Table 5 displays the formal questionnaire after review. The number 1–5 is used to indicate its degree. A questionnaire is designed by summarizing the knowledge points of other research literature (Zhao and Zhao, 2021).

Through the analysis of the various variables in Table 1, under the premise of ensuring the accuracy and reliability of the overall model, Figure 8 displays a schematic diagram of the model.

Overall, 300 questionnaires are distributed to classroom classes through online course software and offline colleges for 1 week’s survey and feedback. A week later, 291 questionnaires are collected, and the incomplete and damaged parts are discarded. Overall, 260 valid questionnaires are collected, and the quantity meet the sample requirements. Table 2 shows the information statistics of the respondents.

**TABLE 2:** Statistics of objects.

Category	Project	Quantity
Gender	Male	135
	Female	125
Age	18–23	186
	24–30	58
	Over 30	16
Academic degree	Associate degree	43
	Bachelor degree	168
	Master’s degree	43
	Doctor’s degree	6
Major	Liberal arts	62
	Science and engineering	165
	Other	33

After the basic information of 260 questionnaires is counted, the survey results of entrepreneurial behavior choice are analyzed. SPSS statistical software and Structural Equation Modeling (SEM) statistical data analysis method are used to analyze the results.

The reason for using SEM data analysis method is that the search performance of the search engine used in SEM analysis method is stronger than that of the Partial Lease Square (PLS), and SEM can convert data into results with higher conversion rate; moreover, the analysis structure of SEM analysis method is a three-dimensional and multi-level form, which can better show the driving force analysis of equipment. This three-dimensional analysis method is more in line with human thinking, which cannot be achieved by traditional PLS analysis at present; next, SEM analysis model can analyze the data attributes that cannot be measured directly, such as abstract data such as user loyalty and satisfaction; finally, SEM analysis model can cause and effect the attributes of each data, so that different levels of data can be compared at the unified level.

SEM comprehensive fitting index is used to evaluate the support of observation data and conceptual model. The value range of the model is between 0 and 1. The closer the value is to 0, the better the observed data fit the model. According to the general standard, it needs to be less than 0.1. The observed data can relatively fit with the model when the value is < 0.05, the observed data can well fit with the model when the value is < 0.01, the observed data and the model are quite well fitted when the value is < 0.08. The value range of other fitting indexes is also between 0 and 1. It is generally believed that the quality of a model should not be evaluated by one, but by multiple indexes.

### Harman Single Factor Test

The basic hypothesis of Harman’s single factor test is that if there is method variation, an unrotated factor will be precipitated when exploratory factor analysis is carried out on all items containing all research constructs, and this common factor explains most of the variation. At present, the more common method is to use confirmatory factor analysis and set the common factor number as 1 to accurately test the hypothesis that “a single factor explains all variations.” Harman’s single factor method is not the best but the most commonly used method. The characteristic of this method is to detect the effect of the result when the source of the common method deviation is not clear. If the result effect is significant, another method can be adopted for statistical control. As the conclusion of this example, the chi square of the model is not significant. It can be considered that the effect of measurement error including common method deviation is not significant and will not have a serious impact on the research conclusion. If the test results are significant and explain a large amount of variation, does it mean that the common method deviation is significant? The answer is No. Perhaps the research variables themselves contain a meaningful common structure, which requires researchers to make scientific judgment.

### Adaptive Learning Method

Adaptive learning is a means of educational science and technology. It provides independent help suitable for each student and has real-time interaction with students in reality. It requires various forms of learning resources, including text, image, sound, video and so on, in order to meet the needs of various learners. Adaptive learning style gives students strong autonomy in learning activities, and teachers only provide guidance. Adaptive learning reconstructs the knowledge acquisition mode. Students no longer rely too much on teachers’ explanation and practice in traditional teaching, but carry out effective learning through interaction with the learning environment, so as to acquire and construct their own knowledge. It regards each learner as a VIP and provides the support of learning programs, computer technology, multimedia, artificial intelligence, communication and other high and new technologies only suitable for the learner. Inspiration: in the classroom, it is essential to learn to set a good learning driven situation according to students’ psychology and future core literacy training, mobilize learning curiosity, create cognitive conflict, be student-oriented, construct their cognitive framework, guide and inspire their thinking.

Adaptive learning generally has three steps. First, it is necessary to establish an appropriate knowledge map according to the learned knowledge, and label and structure each knowledge point systematically; the second step is to make realistic mapping for the learning users; the third is to calculate the most suitable learning path for specific users through specific algorithms.

Entrepreneurial adaptive learning is a learning method that can significantly improve learning performance. The influencing factors of adaptive learning are different when learning is difficult. Among them, plan setting and reflection and error correction are important ways, which play different roles in the learning of entrepreneurial knowledge. Metacognitive level plays a regulatory and promoting role between entrepreneurial learning tasks and learning when starting a business.

Entrepreneurial learning is independently produced by learning objects under the change of environment. Entrepreneurs plan, implement and evaluate their actions, compare the interests they pursue with the feasibility of the problems they need to solve, and finally adjust the learning path. Entrepreneurial learning is very necessary, especially in the trial and error stage. With the progress of the entrepreneurial process, the entrepreneurial environment is constantly changing. Therefore, in this case, entrepreneurs need to have certain experience advantages in solving problems. Adaptive learning plays a very basic role in the learning process of entrepreneurship.

## Results

### Analysis of Standardized Coefficient of Questionnaire Results

**FIGURE 9:**
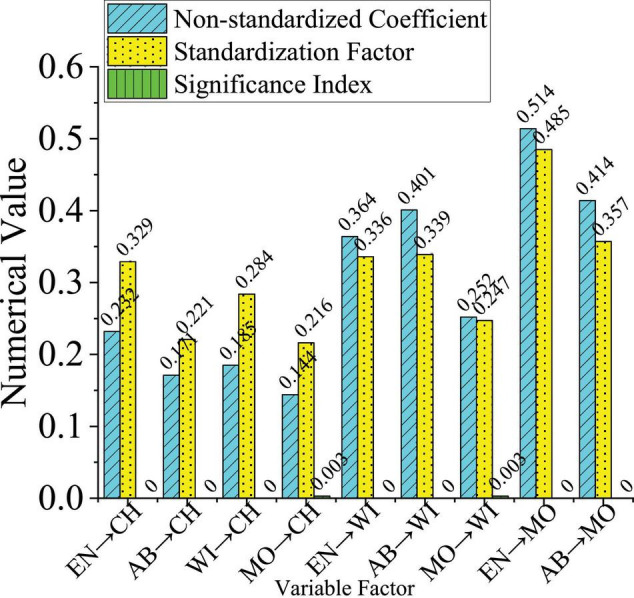
Path coefficient of each variable model (the abscissa in the figure is abbreviated as follows: entrepreneurial environment (EN), entrepreneurial choice (CH), entrepreneurial ability (AB), willingness to act (WI), entrepreneurial motivation (MO), and the arrow indicates that the former affects the latter).

The SEM analysis method is used to make statistics and analysis on the result of the questionnaire on the data analysis software SPSS26.0. The non-standardized regression coefficient of each variable factor, the standard error of estimation parameters and the test statistics are calculated. Table 3 shows the non-standardized results. Then, the data are sorted out, and the standardization factor is shown in Figure 9. When the significance value is less than 0.001, the numerical results are not displayed.

**TABLE 3:** Non-standardized coefficient.

Variable (arrow indicates the influence of the former on the latter)	Effect value (Estimate)	Standard error (S.E.)	Test statistics (C.R.)	Significance (P)
Environment → motivation	0.508	0.79	5.295	
Ability → motivation	0.408	0.84	3.726	
Ability → willingness	0.401	0.81	3.715	
Motivation → willingness	0.248	0.82	1.948	0.003
Environment → willingness	0.374	0.79	3.393	
Willingness → choice	0.186	0.43	2.935	
Motivation → choice	0.142	0.44	1.983	0.003
Environment → choice	0.233	0.44	3.813	
Ability → choice	0.172	0.45	2.785	
Choice → EC1	1.000			
Choice → EC2	1.456	0.138	10.007	
Choice → EC3	1.721	0.149	10.300	
Choice → EC4	1.721	0.142	10.871	
Choice → EC5	1.702	0.148	10.317	
Willingness → BI3	1.000			
Willingness → BI2	1.045	0.058	15.812	
Willingness → BI1	1.075	0.059	16.167	
Motivation → EM4	1.000			
Motivation → EM2	0.879	0.070	11.045	
Motivation → EM1	1.035	0.072	12.736	
Environment → EE5	1.000			
Environment → EE4	1.265	0.078	14.519	
Environment → EE3	1.105	0.080	12.214	
Environment → EE2	1.188	0.076	13.967	
Environment → EE1	1.085	0.079	12.175	
Ability → ERC1	1.000			
Ability → ERC2	0.953	0.075	11.142	
Ability → ERC3	0.877	0.064	12.037	
Ability → ERC4	1.008	0.066	13.441	
Ability → ERC6	1.016	0.079	11.249	
Ability → ERC7	0.829	0.068	10.568	

Figure 9 reveals that for the impact of entrepreneurial environment on entrepreneurial choice, the non-standardization coefficient is 0.232, the standardization factor is 0.329, and the significance index is 0; for the influence of entrepreneurial ability on entrepreneurial choice, the non-standardized coefficient is 0.171, the standardized factor is 0.221, and the significance index is 0; for the impact of behavior intention on entrepreneurial choice, the non-standardized coefficient is 0.185, the standardized factor is 0.284, and the significance index is 0; for the impact of entrepreneurial motivation on entrepreneurial choice, the non-standardized coefficient is 0.144, the standardized factor is 0.215, and the significance index is 0.003; for the impact of entrepreneurial environment on entrepreneurial intention, the non-standardized coefficient is 0.364, the standardized factor is 0.336, and the significance index is 0; for the influence of entrepreneurial ability on entrepreneurial intention, the non-standardized coefficient is 0.401, the standardized factor is 0.339, and the significance index is 0; for the impact of entrepreneurial motivation on entrepreneurial intention, the non-standardized coefficient is 0.252, the standardized factor is 0.247, and the significance index is 0.003; for the impact of entrepreneurial environment on entrepreneurial motivation, the non-standardized coefficient is 0.514, the standardized factor is 0.485, and the significance index is 0; finally, for the impact of entrepreneurial ability on entrepreneurial motivation, the non-standardized coefficient is 0.414, the standardized factor is 0.357, and the significance index is 0. The analysis of Table 3 and Figure 9 reveal that the coefficients of MO, AB, WI and EN affecting entrepreneurial behavior choice are 0.216, 0.221, 0.284, and 0.329, respectively. In addition, MO, AB and EN also affect behavior choice in the form of WI, and their coefficients are (0.247 * 0.216) = 0.070, (0.339 * 0.284) = 0.096, (0.336 * 0.284) = 0.095. AB and EN affect behavior choice through MO, and the coefficient is (0.357 * 0.216) = 0.077, (0.485 * 0.216) = 0.105. Therefore, the influence coefficients of MO, AB, WI and EN on entrepreneurial behavior choice are (0.216 + 0.07) = 0.286, (0.221 + 0.096 + 0.077) = 0.394, 0.284, (0.329 + 0.095 + 0.105) = 0.529, respectively. Therefore, the entrepreneurial ability and the entrepreneurial environment have a great impact on the choice of entrepreneurial behavior, followed by willingness to act and entrepreneurial motivation.

### Judgments of Hypothetical Result

Comparing the above actual standardization factor and significance indicators with the hypothetical prediction to judge whether the hypothesis proposed is tenable. Table 4 shows the details.

**TABLE 4:** Hypothetical judgment.

Hypothesis	Standardization factor	*P*-value judgment	Is it established?
H1: Entrepreneurial motivation has a positive impact on entrepreneurial behavior choice.	0.216	<0.001	Yes
H2: Entrepreneurial ability has a positive impact on the choice of entrepreneurial behavior.	0.221	<0.001	Yes
H3: Willingness to behave has a positive impact on entrepreneurial behavior choice.	0.284	<0.001	Yes
H4: Entrepreneurial environment has a positive impact on entrepreneurial behavior choice.	0.329	<0.05	Yes
H5: Entrepreneurial motivation has a positive impact on entrepreneurial intention.	0.247	<0.05	Yes
H6: Entrepreneurial ability has a positive impact on willingness to behave.	0.339	<0.001	Yes
H7: Entrepreneurial ability has a positive impact on entrepreneurial motivation.	0.357	<0.001	Yes
H8: Entrepreneurial environment has a positive impact on willingness to behave.	0.336	<0.001	Yes
H9: Entrepreneurial environment has a positive impact on entrepreneurial motivation	0.485	<0.001	Yes

**TABLE 5:** Questionnaire on entrepreneurship behavior choice.

Basic Information	Gender A. male B. female
	Age A.18–23 B.24–30 C. over 30
	Educational background A. associate degree B. bachelor degree C. master degree D. doctoral degree
	Major A. liberal arts B. science and engineering C. others
	Number of entrepreneurial experiences A.1 B. 2 C. over 2
	The type of industry you are starting a business or about to start a business. A. agriculture B. architecture C. transportation D. computer E. wholesale and retail F. restaurant G. internet technology H. education I. entertainment J. others
	The model you are starting or about to start. A. independence B. imitation C. innovation
	The scale of entrepreneurship A. 1–10 people B.10–50 people C.50–200 people D. over 200 people
Entrepreneurial behavior choice	Degree of options: 1. very disagree 2. disagree 3. uncertain 4. relatively agree 5. very agree
Entrepreneurial motivation	Recognized by the society 1. 2. 3. 4. 5
	Accumulate wealth 1. 2. 3. 4. 5.
	Realize personal wishes 1. 2. 3. 4. 5.
Entrepreneurial ability	With the support of relatives and friends 1. 2. 3. 4. 5.
	Able to take risks 1. 2. 3. 4. 5.
	Cheerful personality 1. 2. 3. 4. 5.
	Positive attitude toward work and life 1. 2. 3. 4. 5.
	Good business sense 1. 2. 3. 4. 5.
	Clear self-awareness 1. 2. 3. 4. 5.
	Understand the requirements for entrepreneurship 1. 2. 3. 4. 5.
	Strong management ability 1. 2. 3. 4. 5.
	Hope to create performance 1. 2. 3. 4. 5.
Willingness to behave	Change through behavioral choice 1. 2. 3. 4. 5.
	The choice is important for entrepreneurship. 1. 2. 3. 4. 5.
	Changes must be made. 1. 2. 3. 4. 5.
Entrepreneurial environment	The domestic situation is suitable for entrepreneurship. 1. 2. 3. 4. 5.
	Entrepreneurship is not affected by the epidemic. 1. 2. 3. 4. 5.
	With the support of relatives and friends 1. 2. 3. 4. 5.
	Received entrepreneurship education in school 1. 2. 3. 4. 5.
	Entrepreneurship policy helps a lot. 1. 2. 3. 4. 5.
Entrepreneurial choice	Have confidence in choice 1. 2. 3. 4. 5.
	The entrepreneurial process is relatively smooth. 1. 2. 3. 4. 5.
	The entrepreneurial intention is clear. 1. 2. 3. 4. 5.
	Able to make choices easily 1. 2. 3. 4. 5.
	The original intention of starting a business meets expectations. 1. 2. 3. 4. 5.

Table 4 suggests that the hypotheses are all true, so it can be concluded that college students’ entrepreneurial motivation, entrepreneur ability, willingness to behave and entrepreneurial environment will have a significant positive impact on college students’ entrepreneurial choice behavior in the post epidemic era, among which the entrepreneurial environment has the most significant impact on entrepreneurial choice behavior. The judgment results of the above assumptions are as follows. The standardized effect value of H1 (entrepreneurial motivation has a positive impact on entrepreneurial behavior choice) is 0.216, *P* < 0.001, which is true; the standardized effect value of H2 (entrepreneurial ability has a positive impact on the choice of entrepreneurial behavior) is 0.221, *P* < 0.001, which is true; the standardized effect value of H3 (willingness to behave has a positive impact on entrepreneurial behavior choice) is 0.284, *P* < 0.001, which is true; the standardized effect value of H4 (entrepreneurial environment has a positive impact on the choice of entrepreneurial behavior) is 0.329, *P* < 0.05, which is true; the standardized effect value of H5 (entrepreneurial motivation has a positive impact on entrepreneurial intention) is 0.247, *P* < 0.05, which is true; the standardized effect value of H6 (entrepreneurial ability has a positive impact on willingness to behave) is 0.339, *P* < 0.001, which is true; the standardized effect value of H7 (entrepreneurial ability has a positive impact on entrepreneurial motivation) is 0.357, *P* < 0.001, which is true; the standardized effect value of H8 (entrepreneurial environment has a positive impact on willingness to behave) is 0.336, *P* < 0.001, which is true; the standardized effect value of H9 (entrepreneurial environment has a positive impact on entrepreneurial motivation) is 0.485, *P* < 0.001, which is true.

## Discussion

First, the influencing factors of college students’ entrepreneurship under epidemic situation are analyzed by collecting relevant literature. Then, a questionnaire about entrepreneurial environment and entrepreneurial ability of college students who have started or will start a business is made. Finally, the reliability and validity of the survey results are tested by using data analysis software SPSS and analysis algorithm SEM to verify the correlation among variables. The research innovation lies in the analysis of the influencing factors of entrepreneurs’ entrepreneurial behavior, and the coefficients of entrepreneurial motivation, entrepreneurial ability, behavioral willingness and entrepreneurial environment affecting the choice of entrepreneurial behavior are obtained; motivation, ability and environment also affect the coefficient of behavior choice in the form of behavior intention; entrepreneurial ability and environment affect the coefficient of behavior choice through motivation It is concluded that college students’ entrepreneurial motivation, entrepreneur ability, behavior intention and entrepreneurial environment will exert a significant positive impact on college students’ entrepreneurial choice behavior in the post epidemic era. Among them, the entrepreneurial environment has the most significant impact on entrepreneurial choice behavior. Ratten and Jones (2021) studied the relationship between entrepreneurship education and COVID-19 to help understand the future research and practice path. Several hypotheses changed due to COVID-19 and how entrepreneurship education is needed to help solve the entrepreneurship under pandemic are put forward. By considering entrepreneurship education as a whole process from the perspective of entrepreneurial stakeholders, how to carry out the response mechanism including recovery and change can be analyzed deeply. This enables people to regard the COVID-19 crisis as an opportunity to make more people focus more on the importance of entrepreneurship education to society (Ratten and Jones, 2021). Kawamorita et al. (2020) studied the relationship among college, industry, government, society and environment based on entrepreneurial intention. They believed that during the COVID-19 pandemic, they face multiple challenges, and the ecosystem needs their greater attention. The findings reveal the main challenges faced by entrepreneurial colleges and their related potential responses (Kawamorita et al., 2020). The result of the two studies are compared with that of this exploration. It is found that the conclusion is similar, that is, COVID-19 has a certain impact on the entrepreneurial intention of college students. In particular, the impact of entrepreneurial motivation and entrepreneurial environment on entrepreneurial intention further proves the correctness of the research content of this exploration. The entrepreneurial environment has the greatest impact on the choice of entrepreneurial behavior. Therefore, to increase college students’ entrepreneurial willingness and motivation and enhance their entrepreneurial self-confidence, the government, society, colleges and families should strengthen the active guidance of students’ entrepreneurial choice, create a good entrepreneurial environment and cultivate their entrepreneurial ability, to better cope with the impact of the post epidemic era on the economic system. Since the investigation is conducted after the outbreak, the sample size is small, which is not enough to support the entrepreneurial phenomenon of the whole society. In the future research, the scope of sample cities and the number of subjects will be expanded; moreover, the comprehensiveness and rationality of the questionnaire need to be further improved, and the variables of various influencing factors need to be subdivided and expanded. For example, the entrepreneurial environment can be subdivided into government, society, school and family environment. It is hoped that there will be a more in-depth discussion.

## Conclusion

Based on the epidemic era, this exploration aims to study the influencing factors of college students’ behavior choices in the process of entrepreneurship. First, relevant theoretical concepts are found and learned, the variables that affect the choice of entrepreneurial behavior are summarized and sorted out, and the assumptions about the results are made. Then, a questionnaire on the entrepreneurial motivation, entrepreneurial ability, willingness to behave and entrepreneurial environment of college students who have started or will start a business is made. Besides, the model is established based on the structural equation. Through the test, the degree of interaction among various variables is obtained, and it is proved that these variable factors have a positive impact on the choice of entrepreneurial behavior. The results are consistent with the prediction, which proves the correctness of the hypothesis. According to the empirical results, the analysis shows that in the epidemic era, the economic system has been affected and the entrepreneurial environment has changed greatly. College students will consider more environmental factors when starting a business, such as economic conditions, social environment, school environment and family environment. The government, society, colleges and families should strengthen the active guidance of students’ entrepreneurial choice, create a good entrepreneurial environment and cultivate entrepreneurial ability, so as to better deal with the impact of the post epidemic era on the economic system. The practical significance of the conclusion is to provide some entrepreneurial ideas for college students who are going to start a business. Entrepreneurship needs appropriate entrepreneurial objectives and firm entrepreneurial motivation. Besides, it also needs to choose whether to start a business according to its own entrepreneurial environment. The research limitation is that the number of survey samples is small, which is not enough to support the entrepreneurial phenomenon of the whole society. In the future research, the scope of sample cities and the number of subjects will be expanded; besides, the comprehensiveness and rationality of the questionnaire need to be further improved, and the variables of various influencing factors need to be subdivided and expanded. For example, the entrepreneurial environment is subdivided into government, society, school and family environment. It is hoped that there will be a more in-depth discussion.

## Data Availability Statement

The raw data supporting the conclusions of this article will be made available by the authors, without undue reservation.

## Ethics Statement

The studies involving human participants were reviewed and approved by the Lingnan University Ethics Committee. The patients/participants provided their written informed consent to participate in this study. Written informed consent was obtained from the individual(s) for the publication of any potentially identifiable images or data included in this article.

## Author Contributions

The author confirms being the sole contributor of this work and has approved it for publication.

## Conflict of Interest

The author declares that the research was conducted in the absence of any commercial or financial relationships that could be construed as a potential conflict of interest.

## Publisher’s Note

All claims expressed in this article are solely those of the authors and do not necessarily represent those of their affiliated organizations, or those of the publisher, the editors and the reviewers. Any product that may be evaluated in this article, or claim that may be made by its manufacturer, is not guaranteed or endorsed by the publisher.
